# Metallacarboranes as tunable redox potential electrochemical indicators for screening of gene mutation†
†Electronic supplementary information (ESI) available. See DOI: 10.1039/c6sc01567k


**DOI:** 10.1039/c6sc01567k

**Published:** 2016-06-08

**Authors:** Tania García-Mendiola, Victoria Bayon-Pizarro, Adnana Zaulet, Isabel Fuentes, Félix Pariente, Francesc Teixidor, Clara Viñas, Encarnación Lorenzo

**Affiliations:** a Departamento Química Analítica y Análisis Instrumental , Universidad Autónoma de Madrid , Spain . Email: encarnacion.lorenzo@uam.es; b Instituto Madrileño de Estudios Avanzados (IMDEA) Nanociencia , Spain; c Institut de Ciència de Materials de Barcelona (ICMAB-CSIC) , Campus UAB, 08193 Bellaterra , Barcelona , Spain . Email: clara@icmab.es

## Abstract

The substitution of hydrogen with chlorine in the metallacarborane [3,3-Fe(1,2-*closo*-C_2_B_9_H_11_)_2_]^–^ cluster modulates the formal potential of the Fe^3+^/Fe^2+^ redox couple, shifting it to a more positive value. Hence, very similar redox probes with a wide range of formal potentials, ranging from negative to positive values, are available. Thus, we have achieved the synthesis and studied the electrochemical behaviour of the sodium salt of [3,3-Fe(8,9,12-Cl_3_-1,2-*closo*-C_2_B_9_H_8_)_2_]^–^ in aqueous media. This strategy allows tuning of the redox potential of the [3,3-Fe(1,2-*closo*-C_2_B_9_H_11_)_2_]^–^ framework with a minor change in its shape and dimensions. We also describe the interaction of the prepared [3,3-Fe(8,9,12-Cl_3_-1,2-*closo*-C_2_B_9_H_8_)_2_]^–^ and the pristine [3,3-Fe(1,2-*closo*-C_2_B_9_H_11_)_2_]^–^ with DNA. These studies have been carried out not only with DNA in solution but also with DNA immobilized on screen-printed gold electrodes. The results obtained point to a strong interaction between the metallacarboranes and DNA, to a different extent with single stranded DNA (ssDNA) compared to double stranded DNA (dsDNA). This property makes them selective and wide-ranging potential electrochemical indicators of hybridization. The suitability of these new redox indicators for selective DNA biosensor development has been probed by the direct detection of two different mutations associated with cystic fibrosis in PCR amplicons extracted from blood cells.

## Abstract 2

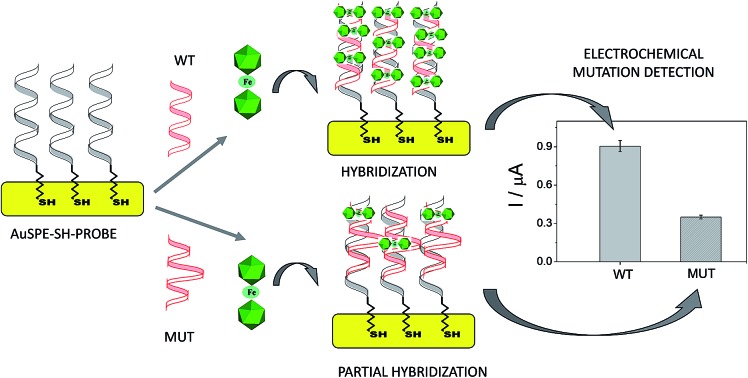
The electrochemical behaviour of metallocarborane sodium salts and their interactions with DNA have been studied. They are shown to be selective redox indicators with wide-ranging potential as electrochemical indicators in DNA biosensors.

## Introduction

The detection of specific DNA sequences provides the fundamental basis for monitoring a wide variety of viral infections, as well as genetic and infectious diseases. DNA biosensors based on nucleic acid recognition processes have received considerable attention in rapid and inexpensive DNA assays.^1^,^2^ Different techniques including fluorescence, surface plasmon resonance, quartz crystal microbalance measurements and electrochemistry have been employed to detect DNA hybridization. Among them, electrochemical transducers are powerful tools for interfacing DNA recognition at the molecular level and converting the hybridization event into an analytical signal.^3^–^6^ They offer attractive advantages such as high sensitivity, low cost and minimal power requirements. Thus, electrochemical DNA biosensors represent a dynamic research area focused on the development of point-of-care tests.^7^ These devices rely on the conversion of DNA base pair recognition events into a useful electrical signal, using either a label-free or label-based method.^8^ The label free method based on the use of an electroactive hybridization indicator is one of the most attractive owing to its simplicity. In this case, the conversion of the hybridization/recognition event into a measurable electrochemical signal is achieved by using an electroactive compound that interacts with single and double stranded DNA to different extents.^2^,^4^ Recently, small molecules and transition metal complexes, generally cations,^9^–^13^ have been used as electrochemical hybridization indicators. However, not all those proposed are sensitive enough for direct single-nucleotide polymorphism (SNP) genotyping. Hence, the study of the interaction of new molecules with DNA in order to design new drugs and diagnostic reagents still remains a formidable task.

**Fig. 1:**
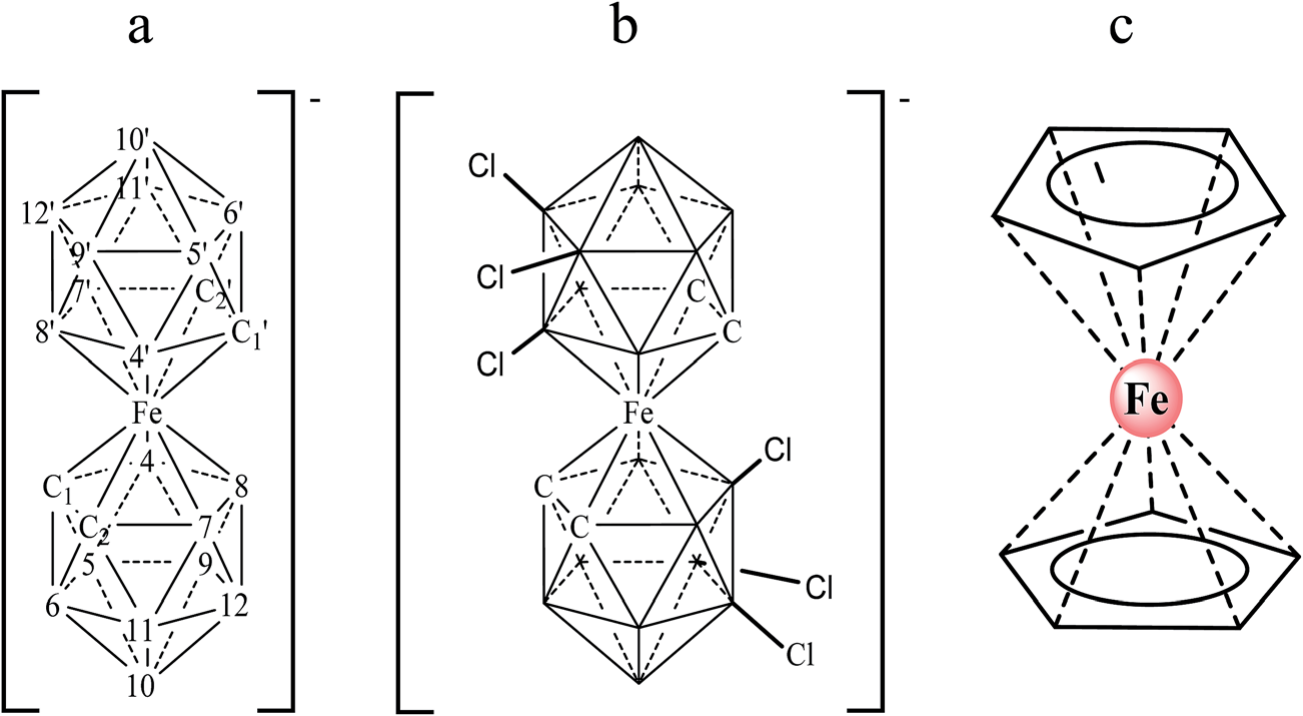
Chemical structures of the anionic metallacarboranes [3,3′-Fe(1,2-*closo*-C_2_B_9_H_11_)_2_]^–^ (a) and [3,3′-Fe(8,9,12-Cl_3_-1,2-*closo*-C_2_B_9_H_8_)_2_]^–^ (b) with their cluster vertex numbering; neutral ferrocene (c).

Metallacarbaboranes (or metallacarboranes) [3,3′-M(1,2-*closo*-C_2_B_9_H_11_)_2_]^*z*–^ are a class of inorganic polyhedral clusters containing carbon, boron, hydrogen, and metal atoms in various combinations.^14^ A typical metallacarborane is a sandwich of two [C_2_B_9_H_11_]^2–^ (dicarbollide) clusters with a metal ion in the center. Fig. 1 displays the [3,3′-Fe(1,2-*closo*-C_2_B_9_H_11_)_2_]^–^ and [3,3′-Fe(8,9,12-Cl_3_-1,2-*closo*-C_2_B_9_H_8_)_2_]^–^ platforms with their cluster vertex numbering. They are metal sandwich complexes similar to ferrocene (see Fig. 1) and have a good reversible electroactive couple, Fe^3+^/Fe^2+^, but unlike ferrocene they are water-soluble. Metallacarboranes are becoming a subject of growing interest to the broad chemical community owing to their unique combination of features and properties, including the rigidity of the cages and their relative rotary motion, their hydrophobicity, and their chemical and thermal stability due to delocalized charge. The inclusion of carbon atoms in the boron framework causes a differential reactivity of the cluster with either acidic (carbon-bound) or hydridic (boron-bound) hydrogen atoms. Moreover, changing the metals further increases the variability, influencing, for instance, the charge or redox properties. Altogether, a broad range of interactions can be formed between metallacarboranes and their neighboring molecules. For example, the B–X bond (X = halogen) in boron clusters is quite stable to reduction and this could be used as an advantage for the redox tuning of boron clusters. Indeed, it has been demonstrated that the incorporation of Cl or I in [3,3′-Co(1,2-*closo*-C_2_B_9_H_11_)_2_]^–^ gradually and systematically shifts the formal potential of Co^3+^/Co^2+^ to positive values.^15^ Thus, the redox potential of a platform can be tuned with a minor change in its shape and dimensions so as to adjust it to a specific purpose.

In addition, [3,3′-Co(1,2-*closo*-C_2_B_9_H_11_)_2_]^–^ and [3,3′-Co(8-I-1,2-*closo*-C_2_B_9_H_10_)_2_]^–^ have proved to have an extraordinary capacity for self-assembly in water.^16^,^17^ This has been demonstrated by the formation of vesicles and micelles for [3,3′-Co(1,2-*closo*-C_2_B_9_H_11_)_2_]^–^ or vesicles and lamellae for [3,3′-Co(8-I-1,2-*closo*-C_2_B_9_H_10_)_2_]^–^, in this order, on increasing the concentration of the cobaltabisdicarbollide. Therefore, it was expected that (i) due to the great tendency to adhere to surfaces through hydrogen or dihydrogen bonds,^18^ and (ii) due to the self assembly capacity, a large multiplier effect could occur at defined potentials.

In spite of these properties, few studies related to DNA and metallacarboranes can be found in the literature. Lesnikowski *et al.* have reported the use of metallacarborane units covalently linked to an oligonucleotide DNA strand as an active label method for the electrochemical detection of DNA hybridization,^19^–^21^ but to date no one has studied their interaction with DNA and their use as free redox hybridization indicators. These compounds may exhibit interesting interactions with DNA; they are anions, and interactions with DNA have usually been described for cations.

In this work, we describe the interaction of two ferrabisdicarbollide species, [Na·2.5H_2_O][3,3′-Fe(1,2-*closo*-C_2_B_9_H_11_)_2_] (denoted Na[FESANE] in the text) and its hexachlorinated derivative [Na·2.5H_2_O][3,3′-Fe(8,9,12-Cl_3_-1,2-*closo*-C_2_B_9_H_8_)_2_] (denoted Na[Cl_6_-FESANE]), with DNA, and their application as negative and positive potential redox indicators for direct gene mutation detection in PCR amplicons extracted from blood cells.

## Results and discussion

### Synthesis and electrochemical properties of the metallacarboranes

**Fig. 2:**
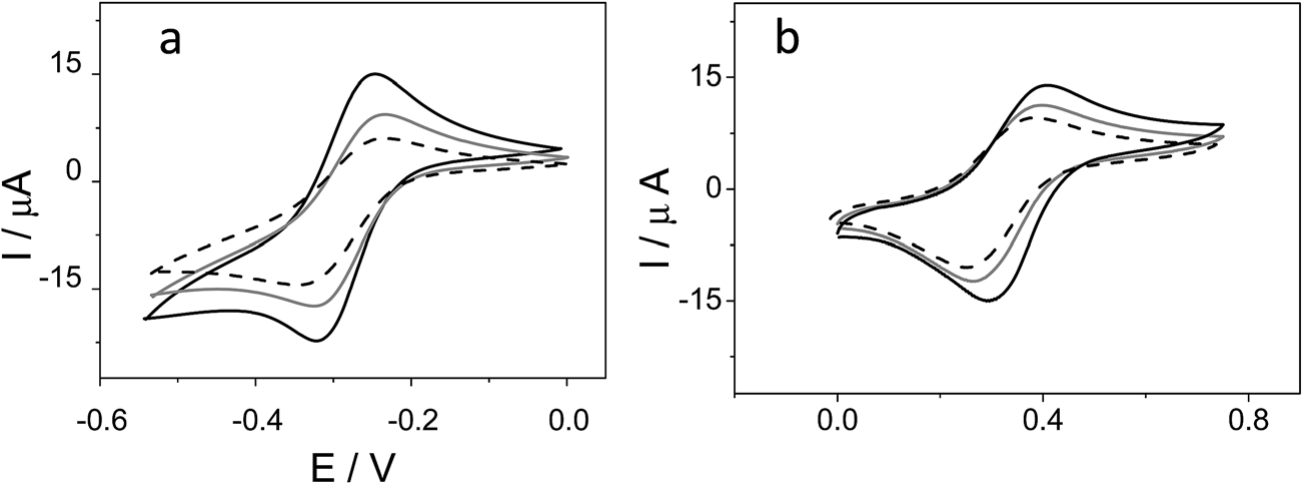
(a) Cyclic voltammograms of 1.0 mM Na[FESANE] in 0.1 M PB pH 7.0 solution at a bare AuSPE (black curve), a calf thymus double stranded DNA AuSPE (CT-dsDNA/AuSPE; grey curve) and a calf thymus single stranded DNA AuSPE (CT-ssDNA/AuSPE; dashed curve). (b) The same experiments for Na[Cl_6_-FESANE].

Ferrocene, with its high stability, is the archetype of sandwich compounds in which a metal is bound in a hapto manner to two arene ligands.^22^ Ferrocene and the metallacarborane sandwich clusters [3,3′-M(1,2-*closo*-C_2_B_9_H_11_)_2_]^*z*–^ (Fig. 1) are air stable.^23^ The substitution of some hydrogen atoms for halogens leads to the halogenated derivatives of pristine [3,3′-M(1,2-*closo*-C_2_B_9_H_11_)_2_]^*z*–^.^15^,^24^–^26^ Similarly to ferrocene, [3,3′-M(1,2-*closo*-C_2_B_9_H_11_)_2_]^*z*–^ species are reversibly electroactive,^27^ but in contrast to ferrocene they are anions whose charge is delocalized through the large molecular volume.^28^ Dehydrochlorination and dehydroiodination on the [3,3′-Co(1,2-*closo*-C_2_B_9_H_11_)_2_]^–^ platform causes an anodic shift of the formal potential (*E*^0^′) of the associated redox couple.^15^,^25^,^26^ The equivalent [3,3′-Fe(1,2-*closo*-C_2_B_9_H_11_)_2_]^–^ platform, [FESANE]^–^, is also susceptible to dehydrochlorination, giving rise to [3,3-Fe(8,9,12-Cl_3_-1,2-*closo*-C_2_B_9_H_8_)_2_]^–^. We have studied the electrochemical behaviour of these two metallacarboranes. Fig. 2 shows cyclic voltammograms (CVs) at 100 mV s^–1^ for 1.0 mM Na[FESANE] (a) and Na[Cl_6_-FESANE] (b) at a bare gold screen-printed electrode (AuSPE) using 0.1 M phosphate buffer (PB) pH 7.0 solution as the supporting electrolyte (black curve). In the case of [FESANE]^–^, it can be seen that a pair of redox peaks, which we ascribe to the metal center (Fe), appear at an *E*^0^′ value of –0.283 V with a peak potential separation (Δ*E*_p_) of 74 mV. In the case of [Cl_6_-FESANE]^–^, a very similar CV response is obtained, but at an *E*^0^′ value of 0.348 V. This shift of about +0.6 V is due to the hydrogen cluster substitution with chlorine groups. The presence of B–Cl bonds in the cluster causes the shift in *E*^0^′, as was previously observed for [3,3′-Co(1,2-*closo*-C_2_B_9_H_11_)_2_]^–^.^25^ Hence, the dehydrochlorination of the cluster triggers the redox potential of the complex to be used for a specific purpose. In this work, the inclusion of six chlorine groups in the [FESANE]^–^ framework allows the preparation of platforms where the redox couple Fe^3+^/Fe^2+^ has a positive or negative formal potential for [FESANE]^–^ and [Cl_6_-FESANE]^–^, respectively.

In the case of [FESANE]^–^, the Δ*E* value is close to the value of 60 mV expected for a freely diffusing one-electron reversible redox process. However, in the case of [Cl_6_-FESANE]^–^, the Δ*E* value is slightly higher, suggesting some limitations in the charge-transfer kinetics, probably due to the presence of the chlorine groups.

In both cases, at a higher ionic strength (0.1 M PB pH 7.0 solution + 0.4 M NaCl) a slightly more reversible shape but similar *E*^0^′ and Δ*E*_p_ values were observed (see Fig. 1 of ESI†). Based on this result one would assume that there are not severe limitations in the charge–transfer kinetics due to the solution resistance. Moreover, the cathodic peak current was proportional to the square root of the scan rate (*v*^1/2^), as expected for a process controlled by diffusion.

### Interaction with DNA

As we have previously mentioned these compounds may exhibit interesting interactions with DNA. Hence, we focused our attention on studying the interaction of [FESANE]^–^ and [Cl_6_-FESANE]^–^ with DNA either in solution or on the electrode surface.

#### Melting curves

**Fig. 3:**
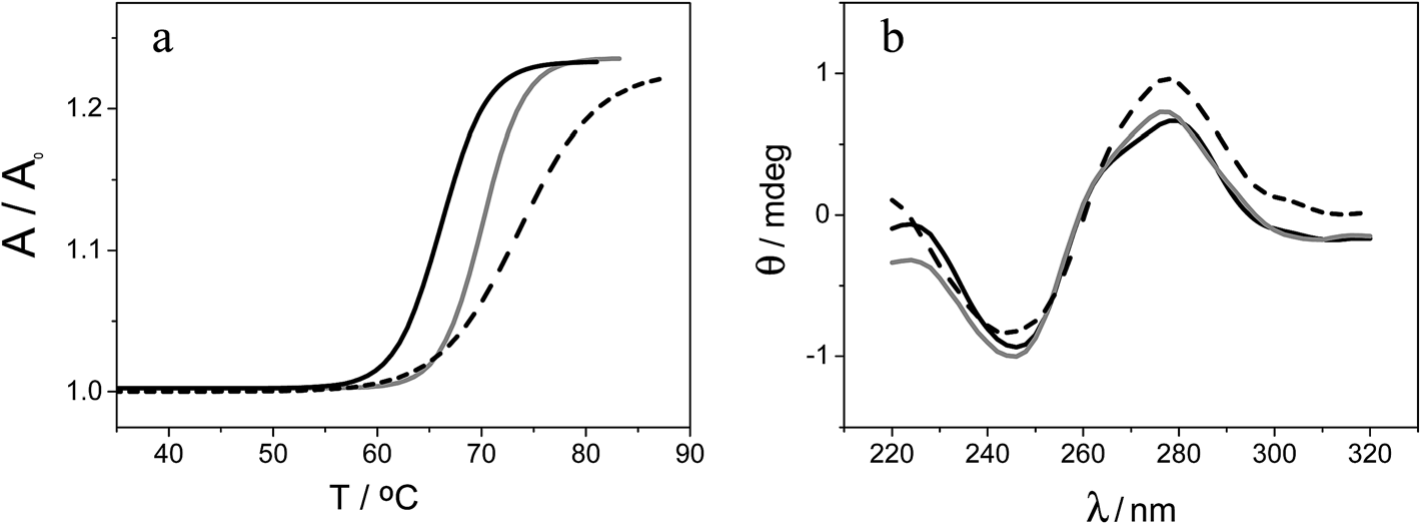
(a) Melting curves of CT-dsDNA (80 μM) in the absence (black curve) and in the presence of 8 μM Na[FESANE] (grey curve) and Na[Cl_6_-FESANE] (dashed curve). (b) Circular dichroism spectra of CT-dsDNA (80 μM) in the absence (black curve) and in the presence of 8 μM Na[FESANE] (grey curve) and Na[Cl_6_-FESANE] (dashed curve).

The DNA double helix resembles a spiral staircase, in which the handrails (strands) are stabilized by hydrogen bonds between base pairs. The interaction of small molecules with the double-helix breaks down the hydrogen bonds between complementary base pairs causing separation of the two polynucleotide strands. The melting temperature (*T*_m_) of DNA is the temperature at which the double helical structure is lost.^29^ The thermodynamics of melting may show interesting interactions. Such experiments could elucidate the interactions of small molecules with the double helix. For example, when a given molecule intercalates into the double stranded DNA, the stability of the double-helix improves and, as a result, the melting temperature increases by about 5–8 °C.^30^ Non-intercalative binding causes no obvious increase in *T*_m_.^31^ We have used this property to analyze the mode of interaction of metallacarboranes with DNA. Such experiments could provide insight into the conformational changes of DNA in the presence of an interacting compound and also information about the interaction strength.^32^ The melting temperature (*T*_m_) was determined by monitoring the absorbance of calf thymus double stranded DNA (CT-dsDNA) (80 μM) at 260 nm as a function of temperature in the absence and in the presence of the metallacarborane (8 μM). One can observe (Fig. 3a) that, in the presence of metallacarborane, the melting temperature exhibits an increase of 4.9 ± 0.2 °C for [FESANE]^–^ and of 7.0 ± 0.3 °C for [Cl_6_-FESANE]^–^, when compared to that of native CT-dsDNA in the absence of metallacarborane. The increase in *T*_m_ observed in the presence of metallacarboranes is consistent with a strong interaction of these compounds with DNA, and suggests that the interaction is mainly intercalative. On the other hand, it is worth noting that the increase is higher in the case of [Cl_6_-FESANE]^–^, pointing to a stronger interaction. A possible explanation for this stability, that will require further tests, is that the existence of six chloro atoms in [Cl_6_-FESANE]^–^ induces a more acidic character for C_c_–H, thus enhancing its capacity to generate hydrogen, dihydrogen or halogen bonds and hence possibly strengthening the interaction between pairs of bases.^33^

In an effort to better understand the interactions of DNA and metallacarboranes Na[FESANE] and Na[Cl_6_-FESANE], the following experiments were conducted.

#### Circular dichroism measurements

Metallacarboranes Na[FESANE] and Na[Cl_6_-FESANE] are achiral compounds, and hence do not exhibit any band in the circular dichroism (CD) spectra. The CT-dsDNA in the B form conformation shows two conservative CD bands, a positive band at 275 nm (due to stacking) and a negative band at 245 nm (due to helicity), in the ultraviolet region.^34^Fig. 3b shows the CD spectra of CT-dsDNA in the absence and in the presence of 8.0 μM metallacarboranes. The positive band at 275 nm was perturbed by the presence of Na[Cl_6_-FESANE], showing an enhancement in the molar ellipticity besides a slight red shift on the band maximum. The molar ellipticity of the negative band, at 245 nm, is shifted towards the positive region in the presence of Na[Cl_6_-FESANE]. In the presence of Na[FESANE], the changes observed in the CD spectrum (see Fig. 3b) are much less noticeable. These observations seem to indicate that the interaction of Na[Cl_6_-FESANE] with DNA is stronger than with Na[FESANE], which agrees well with the melting curve results. Moreover, the changes observed in the DNA CD spectra suggest that the binding of metallacarborane increases the stacking and decreases the helicity of double helical DNA, which is consistent with the intercalative mode of interaction.

#### Absorption titration

**Fig. 4:**
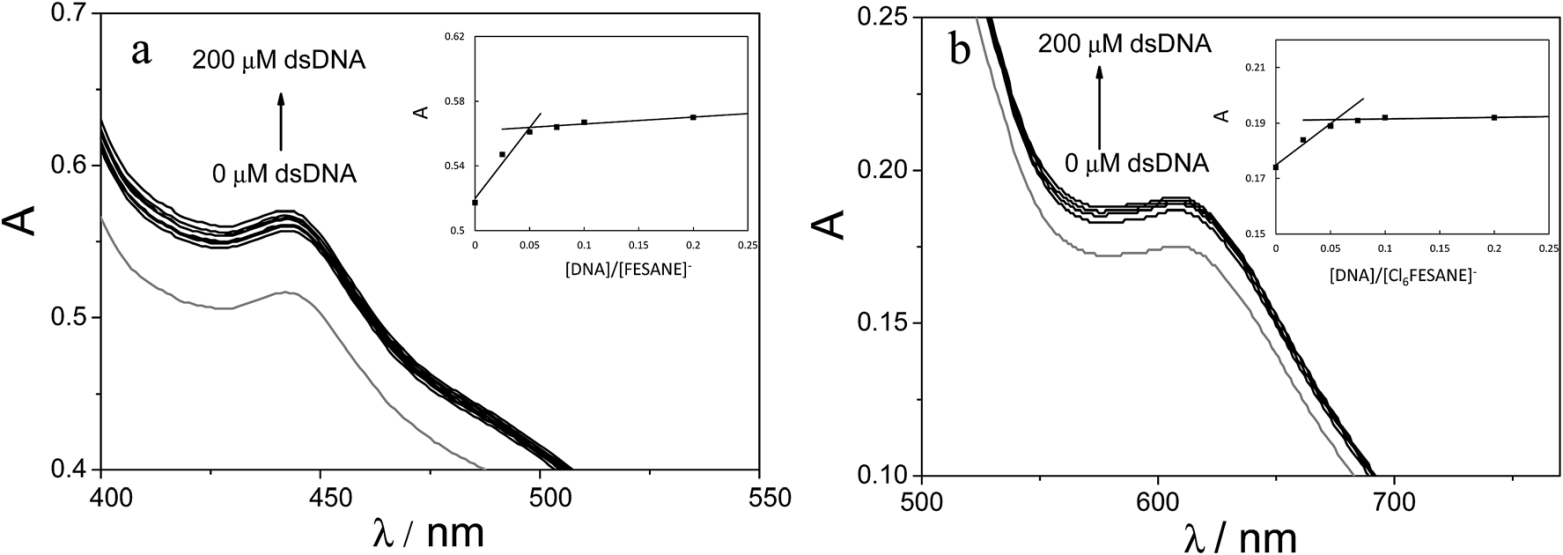
Absorption spectra of 1.0 mM (a) Na[FESANE] and (b) Na[Cl_6_-FESANE] in 0.1 M PB pH 7.0 solution in the absence (grey curve) and in the presence of increasing amounts of CT-dsDNA (from 0 to 200 μM). Inset: absorbance *vs.* [DNA]/Na[FESANE] and absorbance *vs.* [DNA]/Na[Cl_6_-FESANE] plots.

The electronic spectra of metallacarboranes show bands in the UV region (at 270 and 295 nm for Na[FESANE], and 250 and 295 nm for Na[Cl_6_-FESANE]), and in the visible region, at 445 nm for Na[FESANE] and at 620 nm for Na[Cl_6_-FESANE], attributed to the [3,3-Fe (1,2-C_2_B_9_H_11_)_2_]^–^ moiety and to the substituents.^35^Fig. 4 shows the visible absorption spectra of 1.0 mM metallacarboranes in the absence and in the presence of increasing amounts of double stranded calf thymus DNA (CT-dsDNA) in 0.1 M PB pH 7.0 solution. As can be observed, a significant increase in the adsorption band is observed upon addition of double stranded DNA from 0 to 200 μM. It is reported that on studying the interaction of molecules and DNA, hyperchromic effect could be related to an intercalative interaction.^36^ From the titration data plotting of the absorbance *vs.* [DNA]/[metallacarborane] (inset of Fig. 4a and b) it can be concluded that 18 ± 2 molecules of metallacarboranes are bound to a DNA base pair in both cases.

#### Electrochemical experiments with DNA in solution

For these studies, we have used the same conditions as described in Fig. 2. The DNA is electrochemically inactive in the potential range studied. For these electrochemical studies, Na[FESANE] and Na[Cl_6_-FESANE] were kept at a fixed concentration of 1.0 mM while the CT-dsDNA concentration was increased from 0 to 500 μM. In the absence of CT-dsDNA the cyclic voltammograms are the same as those shown by the black curve of Fig. 2. Upon addition of CT-dsDNA, the peak current decreases and the formal potential (*E*^0^′) remains constant for Na[FESANE] and shifts to less positive values for Na[Cl_6_-FESANE] (Fig. 2 of ESI†). This result indicates that metallacarboranes bond to DNA, producing metallacarborane–DNA adducts, which decreases the diffusion coefficient of the unbound metallacarborane species. This is evident from the decrease in the slope of the linear *I*_p_*vs. v*^–1/2^ plot in the presence of CT-dsDNA. Hence, the diffusion coefficients in the absence and in the presence of saturated concentrations of CT-dsDNA (500 μM) were calculated using the Randels–Sevcik equation.^37^ In the absence of DNA, the diffusion coefficients were estimated to be 1.4 ± 0.1 × 10^–6^ and 6.0 ± 0.1 × 10^–7^ cm^2^ s^–1^ for Na[FESANE] and Na[Cl_6_-FESANE], respectively. According to these observations, the presence of the chloro substituents in Na[FESANE] causes a decrease of around two-fold in the diffusion coefficient while in the presence of DNA lower diffusion coefficients of 9.0 ± 0.1 × 10^–7^ and 2.0 ± 0.1 × 10^–7^ cm^2^ s^–1^ were obtained for Na[FESANE] and Na[Cl_6_-FESANE], respectively, as would be expected if metallacarborane–DNA adducts are formed. It is worth mentioning the greater decrease observed in the case of Na[Cl_6_-FESANE], which implies a stronger binding with DNA with respect to [FESANE]^–^, in agreement with the results obtained from the melting curves as well as from the CD studies.

**Fig. 5:**
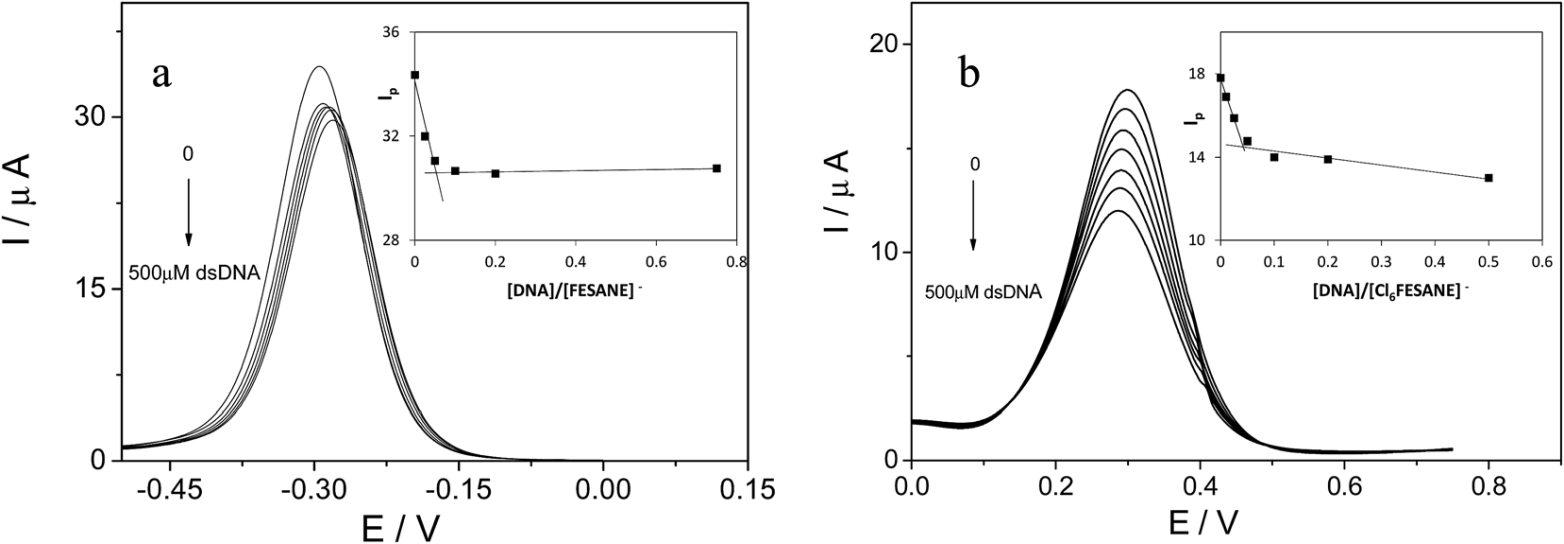
Differential pulse voltammograms of 1.0 mM of Na[FESANE] (a) and Na[Cl_6_-FESANE] (b) in the absence and in the presence of increasing amounts of CT-dsDNA (from 0 to 500 μM). Inset: *I*_p_*vs.* [DNA]/Na[FESANE] and *I*_p_*vs.* [DNA]/Na[Cl_6_-FESANE] plots.

It is well known that the use of pulse voltammetry, such as Differential Pulse Voltammetry (DPV), permits better discrimination between non-faradaic and faradaic current, which is convenient for studying the electrochemical behaviour of redox species that may interact with DNA present in the solution. For this reason, current titrations were performed by means of DPV by keeping the metallacarborane concentration constant while varying the concentration of CT-dsDNA. Fig. 5 shows differential pulse voltammograms for Na[FESANE] and Na[Cl_6_-FESANE]. From the *I*_p_*vs.* [DNA]/[metallacarborane] plot (see inset Fig. 5a and b), it was found that about 20 molecules of metallacarborane are bound to a DNA base pair for both metallacarboranes. This high value, although it agrees well with that obtained from the spectrophotometric titrations, is quite surprising and it can be explained on the grounds of the peculiar structure of [FESANE]^–^ and [Cl_6_-FESANE]^–^, which leads to the possibility of their self-assembly. They are composed of two bulky and highly hydrophobic dicarbollide semicages, each of them bearing two negative charges that “sandwich” a Fe(iii) ion as the polar part. The remaining negative charge is delocalized over the entire [FESANE]^–^ platform that can be represented by the Greek letter θ, as in the case of [3,3′-Co(1,2-*closo*-C_2_B_9_H_11_)_2_]^–^ (denoted [COSANE]^–^).^16^ As can be seen in Fig. 1, because of the charge and θ shape of the [FESANE]^–^ anion, counterions can neither approach it closely enough to reduce the Born energy, nor can the hydrophobic rigid ends deform to partition on one side of the head group, as in double-tailed surfactants.

If [FESANE]^–^ aggregates like [COSANE]^–^,^16^ vesicles with a radius of around ∼20 nm could be expected. In order to corroborate this hypothesis, Dynamic Light Scattering (DLS) experiments were carried out. In these experiments, the concentration of [FESANE]^–^ in the solution was fixed (1.0 mM in water) and the concentration of CT-dsDNA increased from 0 to 900 μM. From the plot of particle size (nm) *versus* CT-dsDNA concentration (Fig. 3 of ESI†), it can be observed that the diameter of the [FESANE]^–^ aggregates is about 39 nm. Upon addition of DNA, a linear increase in particle size up to 63 nm is observed at 150 μM DNA and then a plateau was reached. A new sudden size enlargement appears at a DNA concentration above 300 μM, reaching a size of 75 nm. These results seem to indicate that DNA is surrounding the [FESANE]^–^ aggregates.

#### Electrochemical behaviour of metallacarboranes at DNA modified electrodes

Previous studies have demonstrated that DNA can be adsorbed onto gold screen-printed electrodes (AuSPEs) giving rise to very stable DNA layers.^38^,^39^ Owing to the single component surface structure, such modified electrodes are not only useful for studying the interactions of DNA with other molecules but are also of importance for their potential applications in the development of DNA biosensors. Consequently, AuSPEs were modified with either double-stranded (CT-dsDNA/AuSPEs) or single stranded (CT-ssDNA/AuSPEs) calf thymus DNA and they were employed to study the electrochemical behaviour of the metallacarboranes. The grey curves in Fig. 2 show the CV response of 1.0 mM Na[FESANE] (Fig. 2a) and 1.0 mM Na[Cl_6_-FESANE] (Fig. 2b) solutions at a CT-dsDNA/AuSPE using 0.1 M PB pH 7.0 solution as the supporting electrolyte. It can be observed that there is a decrease in the peak current of the characteristic redox peaks ascribed to the oxidation/reduction of iron and there is a slightly increase in Δ*E*_p_ compared to the CV obtained at the bare AuSPE. Similar results were obtained for CT-ssDNA/AuSPE (dashed curve) but the decrease in current and the increase of Δ*E*_p_ are higher.

**Scheme 1:**
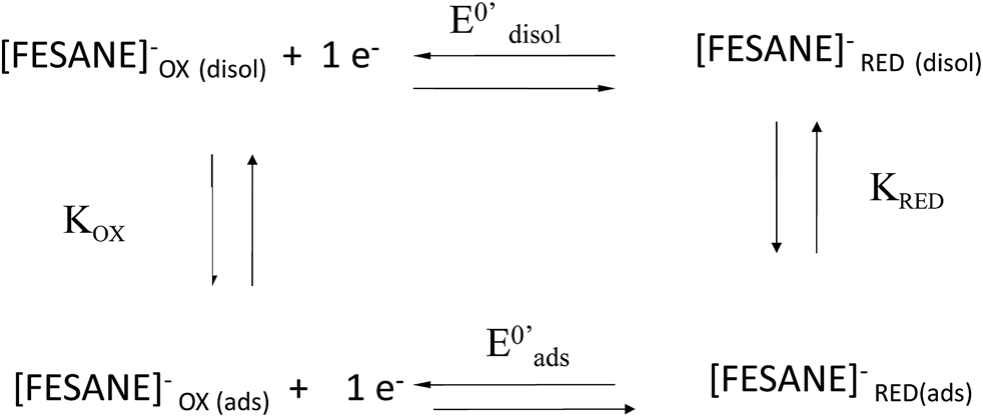
Square scheme of the general process.

In the case of CT-dsDNA/AuSPE the peak current was directly proportional to the scan rate, *v*, which is characteristic of surface-confined redox processes. This seems to indicate that part of the metallacarborane present in the solution is bonding to the CT-dsDNA layer immobilized on the electrode surface. The general process can be described by Scheme 1, similar to the one previously proposed by Bard and Carter.^40^

After repetitive potential cycles, there is an increase in the peak current with no change in *E*^0^, confirming the accumulation of the metallacarborane in the immobilized DNA layer (Fig. 4a of ESI†). If, after repetitive cycling, the electrode was removed from the cell, rinsed with water, and placed in an electrolyte solution containing no metallacarborane, a pair of voltammetric peaks, less defined but very similar to those previously obtained, were observed (Fig. 4b of ESI†). This indicates that the incorporated metallacarborane remained tightly bound to the DNA layer on the electrode surface.

If the ionic strength of the solution increases (0.1 M PB pH 7.0 solution + 0.4 M NaCl), either the shape or the formal potential of the voltammetric waves remain equal. These results confirm that the interaction between CT-dsDNA/AuSPE and the metallacarboranes is largely independent of the ionic strength, which is consistent with intercalation (Fig. 5 of ESI†).

Table 1 displays the *E*^0^′ values of [FESANE]^–^ and [Cl_6_-FESANE]^–^ using a bare AuSPE, a CT-dsDNA/AuSPE and a CT-ssDNA/AuSPE. *E*^0^′ shifted to less negative (in the case of [FESANE]^–^) or less positive (in the case of [Cl_6_-FESANE]^–^) values for a CT-dsDNA/AuSPE compared to those obtained at the bare AuSPE. On the other hand, for CT-ssDNA/AuSPE there is a shift of *E*^0^′ to more negative (in the case of [FESANE]^–^) or less positive (in the case of [Cl_6_-FESANE]^–^) values compared to those obtained at the bare AuSPE. From these shifts (Δ*E*^0^′) and using eqn (1) of the Experimental section, according to the model of Bard and Carter,^40^ the ratio of the equilibrium surface-binding constants (*K*_ox_/*K*_red_) were estimated. For CT-dsDNA/AuSPE the values obtained were 0.76 and 1.73, for [FESANE]^–^ and [Cl_6_-FESANE]^–^, respectively, while at a CT-ssDNA/AuSPE, the equilibrium surface-binding constants were estimated to be 1.14 and 3.35 for [FESANE]^–^ and [Cl_6_-FESANE]^–^, respectively.

**Table 1:** Formal potential (*E*^0^′) of [FESANE]^–^ and [Cl_6_-FESANE]^–^ at a bare AuSPE, a CT-dsDNA and a CT-ssDNA/AuSPE

	AuSPE *E*^0^′ (V)	CT-dsDNA/AuSPE *E*^0^′ (V)	CT-ssDNA/AuSPE *E*^0^′ (V)
[FESANE]^–^	–0.283	–0.276	–0.291
[Cl_6_-FESANE]^–^	0.348	0.334	0.317

The value of 0.76 for *K*_ox_/*K*_red_ obtained in the case of [FESANE]^–^ suggests that the reduced form interacts with the CT-dsDNA backbone more strongly than the oxidized form, which is characteristic of intercalative interactions. However, in the case of [Cl_6_-FESANE]^–^ a *K*_ox_/*K*_red_ value of 1.14 indicates that the oxidized form interacts more strongly than the reduced form, suggesting some electrostatic component in the interaction. This attests the first of the two possibilities given above concerning the role of the [Cl_6_-FESANE]^–^. Nevertheless, the effect of ionic strength is not relevant in the interaction of both clusters with DNA (Fig. 5 of ESI†), which points to the intercalation being predominant.

In the case of CT-ssDNA/AuSPEs, as one would expect, either for [FESANE]^–^ or [Cl_6_-FESANE]^–^ the values of *K*_ox_/*K*_red_ indicate that the oxidized form interacts with the CT-ssDNA backbone more strongly than the reduced form, which agrees well with an electrostatic interaction.

#### DNA sensing

Based on the results described above, the use of both metallacarboranes as electrochemical indicators for applications in DNA sensing was evaluated. For this purpose, in a first approach, a synthetic 25-mer sequence of *Helicobacter pylori* (*H. Pylori*) was employed as a prototype system. *H. Pylori* is a bacterium that can cause digestive illness and even stomach cancer. It has been chosen as a case study due the importance of having a rapid and simple method for its detection, as well as within the framework of developing approaches of broad applicability.

**Scheme 2:**
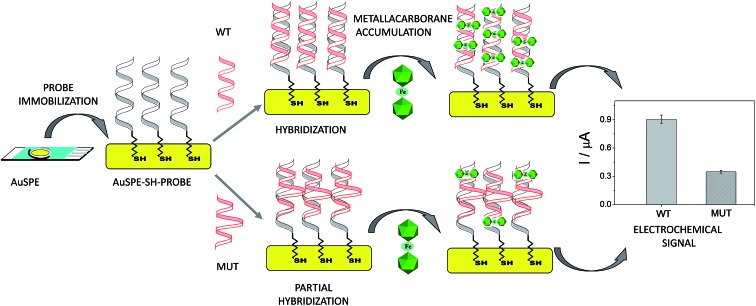
Scheme of the biosensor development.

**Fig. 6:**
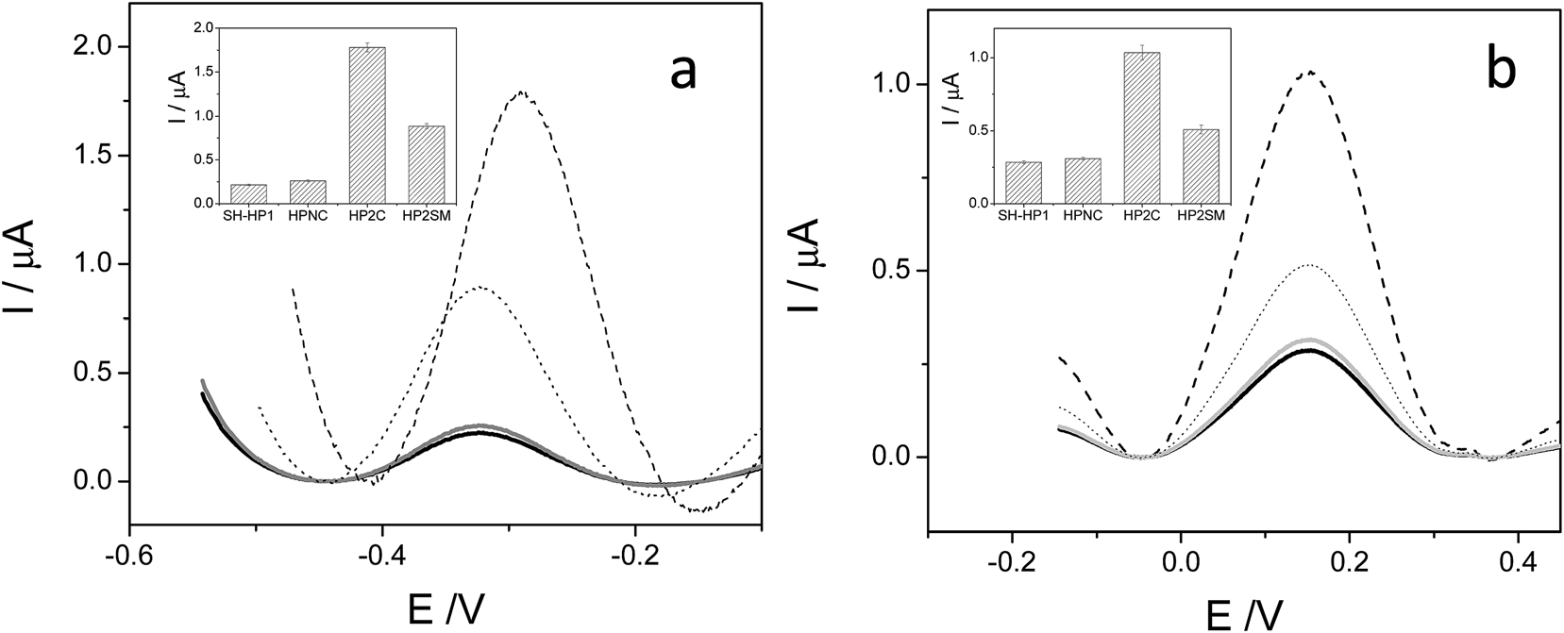
Differential pulse voltammograms of 1.0 mM Na[FESANE] (a) and Na[Cl_6_-FESANE] (b) accumulated on a SH-HP1/AuSPE before (black curve) and after hybridization with a complementary HP2C (dashed curve), non complementary HP2NC (grey curve) and Single Nucleotide Polymorphism (SNP) sequence HP2SNP (dotted curve). Inset: peak current and error bar diagrams of the biosensor response (*N* = 5, RSD less than 5% for all measurements).

A unique 25-mer thiolated sequence of this bacterium (SH-HP1; 10 μl of 40 μM) was chemisorbed onto the AuSPE through the thiol group, with a surface coverage (*σ*) of around 90 pmol cm^–2^, and used as the probe. In the hybridization test, a complementary (HP2C), a non-complementary (HP2NC) and a Single Nucleotide Polymorphism (SNP) sequence (HP2SNP), were selected as the target DNA. Changes in the differential pulse voltammogram peak currents of the clusters accumulated at the probe modified electrode before and after hybridization (see Scheme 2) were obtained and are shown in Fig. 6. As can be seen, hybridization of the probe (SH-HP1) with the complementary HP2C chain in the biosensor recognition layer resulted in a dramatic increase in the DPV response (about 8 and 5 times for [FESANE]^–^ and [Cl_6_-FESANE]^–^, respectively), while virtually no change in current was obtained for the non-complementary sequence (compare the black and grey curves in Fig. 5) either for [FESANE]^–^ or [Cl_6_-FESANE]^–^. The selectivity of the system was probed by hybridization with a SNP target (HP2SNP) sequence. This target will give a distorted double-helix, which may interact with DNA in a different way. Based on this, one would anticipate a different biosensor response when compared to hybridization with a fully complementary target sequence (HP2C). As can be seen, there is a significant decrease (about 50%) in the peak current values compared to those obtained when the SH-HP1/AuSPE electrode was hybridized with its fully complementary sequence, HP2C, (Fig. 5, dashed curve). This diminution in the peak current shall be interpreted as a decrease in the binding constant of the metallacarborane with the distorted helix. However, the peak current obtained when the SNP was present was much higher (about 2 and 4 times for [FESANE]^–^ and [Cl_6_-FESANE]^–^, respectively) than that obtained for a fully non-complementary sequence (see grey curve in Fig. 6). This allows clear discrimination between non-complementary, fully complementary and mismatched base pairs. To our knowledge this is the first time that metallacarboranes have been used for such a purpose.

### Gene mutation detection

Based on these promising results, we decided to go one step further and apply the DNA biosensor to diagnose genetic diseases through the detection of gene mutations associated to such diseases in real DNA PCR amplicons extracted from blood cells. Genetic diseases may be diagnosed by different test categories. Among them, mutation identification by sequencing of genes is the gold standard. However, these methods have serious drawbacks as routine diagnostic tools because of their labour intensity and cost. Hence, new, accurate and facile strategies for the detection of point mutations are therefore absolutely necessary. In this sense, the DNA biosensor could be an alternative to a classical gene assay due to its several advantages, in particular the low cost and simplicity.

As a case study, two different mutations in the cystic fibrosis transmembrane conductance regulator gene (CFTR) were detected. In particular, we chose the F508del (MUTF508del)^41^ and p.Gly542Stop (SNPG542X) mutations, both associated with cystic fibrosis. The F508del mutation consists of a three-nucleotide deletion, which causes the loss of a phenylalanine residue of the CFTR protein,^42^ and the p.Gly542Stop mutation results in a truncated CFTR protein.

In the approach employed, no labelled oligonuclotides are required. Two different thiolated sequences from exons 11 and 12 (named SH-WT_11_ and SH-WT_12_; see Table 3 of the Experimental section), were assembled on the electrode surface, through the thiol group, and hybridized with the corresponding denaturated PCR target, which comprises around 300 bp PCR amplicons of exons 11 and 12 from the CFTR gene, carrying the mutations MUTF508del and SNPG542X associated to cystic fibrosis (see Table 3). As a control, two wild type amplicons, WT_11_ and WT_12_ from exons 11 and 12, respectively, were used. All samples were validated by the suppliers (Instituto de Genética Médica y Molecular de Madrid, Spain) by sequentiation methods.

**Fig. 7:**
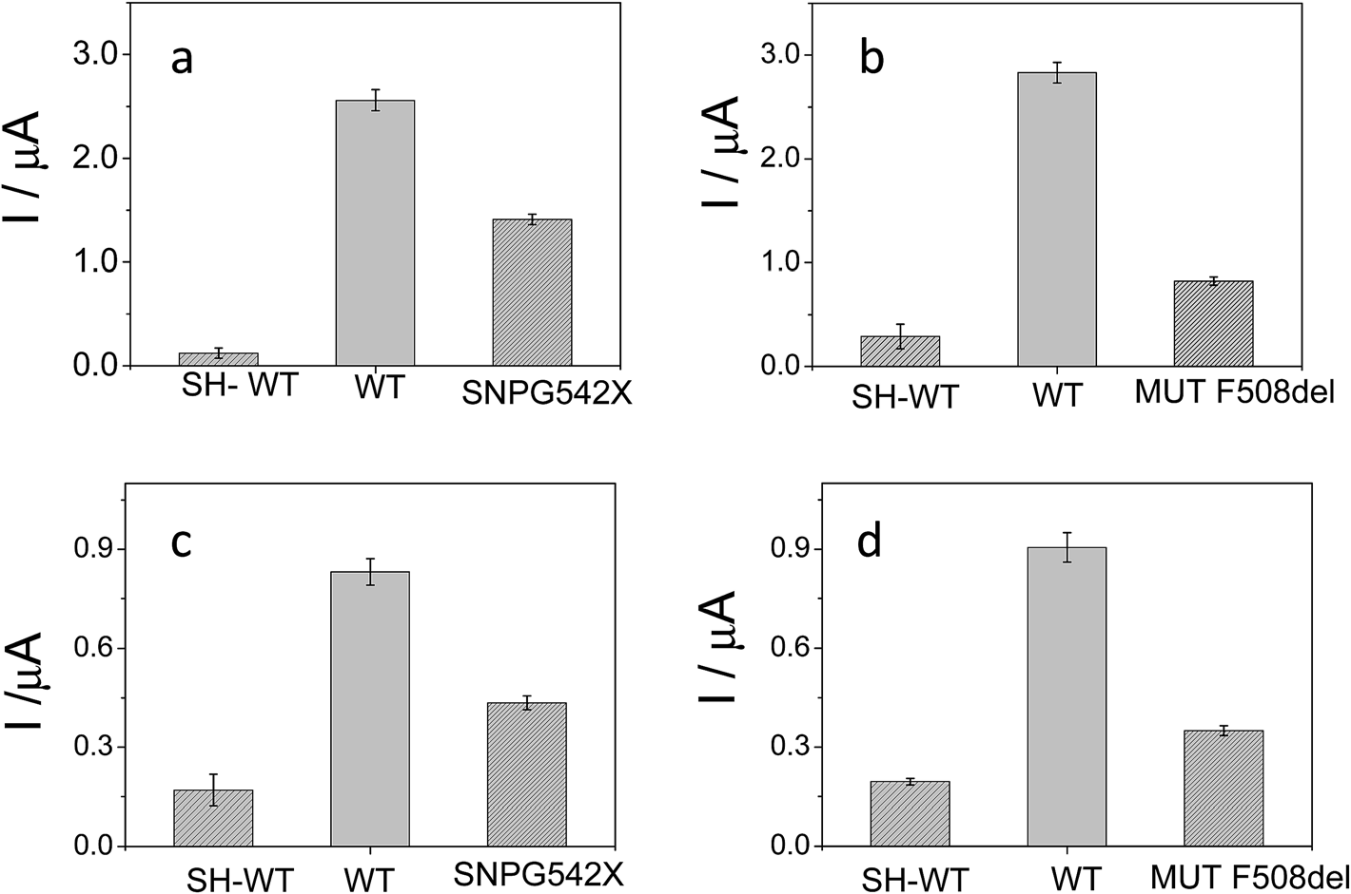
Peak current bar diagrams of the biosensor response before and after hybridization with a solution containing: the control sequence (WT) and mutated sequences, SNPG542X and MUTF508del, using [FESANE]^–^ (a, b) or [Cl_6_-FESANE]^–^ (c, d) as the redox indicator.

The detection relies on the comparison of the voltammetric transduction of the hybridization reaction between the immobilized probe and the target DNA sequences (wild type or mutant) present in the sample. DPV of the metallacarborane accumulated on the double strand DNA layer, formed on the electrode surface after hybridization, was used to obtain the biosensor response. Fig. 7 shows peak current bar diagrams of the biosensor response before and after hybridization with a solution containing: the control sequence (WT) or mutated sequences, SNPG542X and MUTF508del, using [FESANE]^–^ (a, b) or [Cl_6_-FESANE]^–^ (c, d) as the redox indicator. The difference between the signals from the mutated and wild type target DNA allows unambiguous confirmation of the presence of a mutation without the need for the stringent conditions (*i.e.* formamide) usually employed for such a purpose. In addition, it is worth noting that the decrease in the current signal is a slightly higher for SNPG542X than for the MUTF508del samples. In this case the biosensor permits better discrimination between the mutated and wild type samples. This agrees well with the major distorted DNA helix formed after hybridization with the sequence carrying this mutation, which involves a three-nucleotide deletion.

The reproducibility of the developed methodology was evaluated from the response of 5 different biosensors, prepared in the same manner (see Experimental section), to either wild type or mutated target DNA. Reproducible signals were obtained with a relative standard deviation (RSD) of 5% or less in all cases. Hence, it can be concluded that the proposed screening method not only allows DNA detection but also discriminates between wild and mutated samples. These results compare well with those obtained with other more conventional (cationic) redox indicators as the ruthenium complex, pentaamine ruthenium [3-(2-phenanthren-9-yl-vinyl)-pyridine],^12^ or dyes such as Azure A or Safranine,^43^ but metallacarboranes are anionic species. This is the first time that an anion has been employed as the redox indicator of a hybridization event for the detection of gene mutations. Rapid and precise screening of small genetic variations, such as single nucleotide polymorphisms (SNPs) or point mutations as deletions, plays an important role in human diseases. As genetic markers, mutations can be used to trace the generational inheritance patterns associated with specific diseases. As diagnostic markers, point mutations can be used for early cancer^44^ or human disease detection, such as hereditary liver disorders or cystic fibrosis.

## Experimental section

### Chemicals

Potassium nitrate, sodium phosphate and sodium chloride were obtained from Scharlab Company. Double stranded calf thymus DNA (CT-dsDNA) was purchased from Sigma-Aldrich Co. CT-dsDNA stock solutions (nominally 1.0 mg ml^–1^) were prepared in 0.1 M phosphate buffer (PB) pH 7.0 solution. The DNA solutions gave a UV absorbance ratio (*A*_260_/*A*_280_) of about 1.9, indicating that the DNA was free of protein.^45^ The concentration of base pairs (bp) in the DNA was determined by using a molar absorptivity of 6600 M^–1^ at 260 nm.^46^ Single stranded calf thymus DNA (CT-ssDNA) was obtained by boiling in water capped vials containing CT-dsDNA (1.0 mg ml^–1^) in 0.1 M PB pH 7.0 for 30 minutes followed by rapid cooling in an ice bath. The synthetic oligonucleotides (from Sigma-Aldrich Co.) used in this work were 25 or 100-mer sequences from the pathogen bacterium *Helicobacter pylori* (*H. Pylori*) or characteristic DNA sequences from the cystic fibrosis transmembrane conductance regulator (CFTR) gene. Two different types of synthetic oligonucleotides were used – thiolated sequences (SH-HP1 and SH-WT_11_, SH-WT_12_) and non thiolated sequences (HP2C, HP2NC, HP2SNP) that have been used as analytes (see Table 2). Genomic DNA was isolated from peripheral blood leukocytes from cystic fibrosis patients by standardized procedures (commercial Kit Purogene from Qiagen GmbH, Hilden, Germany) as we described before.^12^ The polymerase chain reaction (PCR) samples consisted of wild type (WT_11_, WT_12_) and mutated (MUTF508del and SNPG542X) sequences from gene samples. Synthetic oligonucleotides and PCR amplified sequences of real DNA samples from healthy or cystic fibrosis patients are listed in Tables 2 and 3 respectively. All solutions were prepared just prior to use. Water was purified with a Millipore Milli-Q-System (18.2 MΩ cm).

**Table 2:** Synthetic DNA sequences used in this work

**Synthetic oligonucleotides (25bp)**		
Thiolated probe	5′-HS(CH_2_)_6_-GCGTTCCAAAGGGCAGGATCATTGA	SH-HP1
Complementary sequence	5′-TCAATGATCCTGCCCTTTGGAACGC	HP2C
SNP sequence	5′-TCAATGATCCTACCCTTTGGAAGCG	HP2SNP
Non complementary sequence	5′-GACCGTCGAAGTAAAGGGTTCCATA	HP2NC

**Synthetic oligonucleotides (100bp)**		
Thiolated probe 11	5′-HS(CH_2_)_6_-CTCAGTTTTCCTGGATTATGCCTGGCACCATTAAAGAAAATATCATCTTTGGTGTTTCCTATGATGAATATAGATACAGAAGCGTCATCAAA GCATGCC	SH-WT_11_
Thiolated probe 12	5′-HS(CH_2_)_6_-TTGGTAATAGGACATCTCCAAGTTTGCAGAGAAAGACAATATAGTTCTTGGAGAAGGTGGAATCACACTGAGTGGAGGTCAACGAGCAAGAATTTCTTTA	SH-WT_12_

**Table 3:** PCR amplified sequences from real DNA samples used in this work

PCR samples			
Complementary sequence exon 11	5′_AACCGATTGAATATGGAGCCAAATATATAATTTGGGTAGTGTGAAGGGTTCATATGCATAATCAAAAAGTTTTCACATAGTTTCTTACCTCTTCTAGTTGGCATGCTTTGATGACGCTTCTGTATCTATATTCATCATAGGAAACACCAAAGATGATATTTTCTTTAATGGTGCCAGGCATAATCCAGGAAAACTGAGAACAGAATGAAATTCTTCCACTGTGCTTAATTTTACCCTCTGAAGGCTCCAGTTCTCCCATAATCACCATTAGAAGTGAAGTCTGGAAATAAAACCCATCATTATTAGGTCATTATCAAATCACGCTCAGGATTCACTTGCCTCCAATTATCATCCTAAGCAGAAGTGTATATTC	WT_11_	
Mutated sequence: mutation F508del	5′_AACCGATTGAATATGGAGCCAAATATAATTTGGGTAGTGTGAAGGGTTCATATGCATAATCAAAAAGTTTTCACATAGTTTCTTACCTCTTCTAGTTGGCATGCTTTGATGACGCTTCTGTATCTATATTCATCATAGGAAACACCA___ATGATATTTTCTTTAATGGTGCCAGGCATAATCCAGGAAAACTGAGAACAGAATGAAATTCTTCCACTGTGCTTAATTTTACCCTCTGAAGGCTCCAGTTCTCCCATAATCACCATTAGAAGTGAAGTCTGGAAATAAAACCCATCATTATTAGGTCATTATCAAATCACGCTCAGGATTCACTTGCCTCCAATTATCATCCTAAGCAGAAGTGTATATTC	MUTF508del	
Complementary sequence exon 12	5′_ACTAGCCATAAAACCCCAGGATTTTTTCAATTCCAGAAACAGAATATAAAGCAATAGAGAAATGTCTGTAATTTTTTTACATGAATGACATTTACAGCAAATGCTTGCTAGACCAATAATTAGTTATTCACCTTGCTAAATTCTTGCTCGTTGACCTCCACTCAGTGTGATTCCACCTTCTCCAAGAACTATATTGTCTTTCTCTGCAAACTTGGAGATGTCCTATTACCAAAAATAGAAAATTAGAGAGTCACTTTTAGTATGCTCAATCTGAATTTGAAAGGCACATCTTCCTTCTAATGT	WT_12_	
Mutated sequence: mutation G542X	5′_ACTAGCCATAAAACCCCAGGATTTTTTCAATTCCAGAAACAGAATATAAAGCAATAGAGAAATGTCTGTAATTTTTTTACATGAATGACATTTACAGCAAATGCTTGCTAGACCAATAATTAGTTATTCACCTTGCTAAAGAATTCTTGCTCGTTGACCTCCACTCAGTGTGATTCCACCTTCTC**A[combining low line]**AAGAACTATATTGTCTTTCTCTGCAAACTTGGAGATGTCCTATTACCAAAAATAGAAAATTAGAGAGTCACTTTTAGTATGCTCAATCTGAATTTGAAAGGCACATCTTCCTTCTAATGT	SNPG542X	

[N(CH_3_)_4_][3,3′-Fe(1,2-*closo*-C_2_B_9_H_11_)_2_], [N(CH_3_)_4_][FESANE], was synthesized as reported in the literature.^47^

### Apparatus

UV-visible spectra were recorded using a double beam PharmaSpec UV-1700 series spectrophotometer from Shimadzu Corporation, operating from 200 nm to 800 nm in 1.0 cm quartz cells. A thermostatic bath in conjunction with the spectrophotometer was used to control the cuvette temperature in the measurements. The sequencing of the DNA samples was carried out using a Sanger Sequencher 3730xL (Array 36 cm; POP 7; Applied Biosystems). The final concentration of the DNA sample solutions was determined by UV-vis molecular absorption spectrometry using a Thermo Scientific NanoDrop™ 1000 Spectrophotometer (NanoDrop Technologies). PCR fragments were generated in a BIO-RAD thermal cycler (DNA Engine Tetrad2; Peltier Thermal Cycler; BIO-RAD Laboratories Inc.). Circular dichroism measurements were carried out using a spectropolarimeter J715 from JASCO. Electrochemical measurements were carried out at room temperature in a homemade one-compartment electrochemical cell coupled to an Autolab PGSTAT 30 potentiostat from Eco-Chemie (KM Utrecht, the Netherlands) using the software package GPES 4.9 (General Purpose Elec. Experiments). Integrated screen-printed gold electrodes (4 mm diameter, AuSPEs) from DropSens S.L (Oviedo, Spain) were used as working electrodes. The format of these screen-printed electrodes includes a gold working electrode, a silver pseudo reference electrode and a gold counter electrode. All experiments were carried out by substituting the Ag ink pseudo reference with an external Ag/AgCl reference electrode. The electrodes were connected using a SPE Connector (DropSens S. L.) as the interface. IR spectra were obtained on a PerkinElmer® Universal ATR Accessory spectrophotometer. The ^1^H- and ^1^H{^11^B}-NMR (300.13 MHz), ^13^C{^1^H}-NMR (75.47 MHz) and ^11^B- and ^11^B{^1^H}-NMR (96.29 MHz) spectra were recorded on a Bruker ARX 300 instrument equipped with the appropriate decoupling accessories. All NMR spectra were performed in deuterated acetone solvent at 22 °C. The ^11^B- and ^11^B{^1^H}-NMR shifts were referenced to external BF_3_·OEt_2_, while the ^1^H, ^1^H{^11^B}, and ^13^C{^1^H}-NMR shifts were referenced to SiMe_4_. Chemical shifts are reported in units of parts per million downfield from the reference, and all coupling constants are in Hz. The mass spectra were recorded in the negative ion mode using a Bruker Biflex MALDI-TOF-MS [N_2_ laser; *λ*_exc_ 337 nm (0.5 ns pulses); voltage ion source 20.00 kV (Uis1) and 17.50 kV (Uis2)].

### Procedures

#### Synthesis of [N(CH_3_)_4_][Cl_6_-FESANE]

To a solution of [N(CH_3_)_4_][3,3′-Fe(1,2-*closo*-C_2_B_9_H_11_)_2_] (100 mg, 1.01 mmol) in acetonitrile (3 ml) in a 10 ml round bottomed flask was slowly added SO_2_Cl_2_ (3 ml) drop by drop. The mixture was heated for 1 h at 70 °C. After 5 minutes, all volatiles were removed under reduced pressure and the dark green solid residue was extracted with diethyl ether and water. The mixture was shaken and the two layers were separated. The diethyl ether layer was washed twice with water (2 × 15 ml) and diethyl ether (15 ml). The combined organic layers were dried over MgSO_4_. The filtrate was evaporated and dissolved in the minimum volume of water. An aqueous solution containing an excess of [N(CH_3_)_4_]Cl was added, resulting the formation of a dark green precipitate. This [N(CH_3_)_4_][3,3-Fe(8,9,12-Cl_3_-1,2-*closo*-C_2_B_9_H_8_)_2_] was filtered off and washed several times with water. Yield: 260 mg (83%). UV-vis in acetonitrile at 10^–4^ M: 251 (0.557), 298 (1.561), 338 (1.571), 607 (0.036) nm.

#### Synthesis and characterization of [Na·2.5H_2_O][FESANE] and [Na·2.5H_2_O][Cl_6_-FESANE]

The sodium salts of the [FESANE]^–^ and [Cl_6_-FESANE]^–^ species were obtained by means of cationic exchange resin.

##### General procedure of cationic exchange resin

To get the sodium counterbalancing species, approximately 100 mg of the starting compound, [N(CH_3_)_4_][FESANE] and [N(CH_3_)_4_][Cl_6_-FESANE], which are water-insoluble, were dissolved in a minimum volume of acetonitrile/water (50 : 50). Then each solution was passed repeatedly through a cation exchange resin, previously loaded with sodium. The solvent was finally evaporated. The partial or complete solubilization of the new salts in distilled water and the disappearance of the [NMe_4_]^+^ peaks in the IR and ^1^H NMR spectra was a clear indication that the complete exchange to sodium was successful.

#### Characterization of [Na·2.5H_2_O][FESANE] and [Na·2.5H_2_O][Cl_6_-FESANE]

##### [Na·2.5H_2_O][FESANE]

IR (KBr): *ν* [cm^–1^] = 3590, 3562, 3521 (vs, *ν*_s_(H_2_O)), 3037, 3021 (vs, *ν*_s_(C_c_–H)), 2566, 2521 (vs, *ν*_s_(B–H)), 1636, 1605 (vs, *ν*(H_2_O)). ^1^H{^11^B} NMR: *δ* [ppm] = 69.30 (br s), 45.65 (s, B(10)–H), 39.69 (br s), 1.32 (br s, B–H), –7.8 (br s, B–H). ^11^B NMR: *δ* [ppm] = 103.7 (s, 2B), 21.5 (s, 4B), 0.6 (s, 4B), –31.1 (s, 2B), –403.5 (s, 4B), –453.5 (s, 2B). ^13^C{^1^H} NMR: *δ* [ppm] = –438.19 (br. s, C_c_). MALDI-TOF MS: *m*/*z* (%) = 321.16 [M, 100%]^–^. UV-vis in H_2_O at 2.5 × 10^–5^ M: 271 (2.066) nm.

##### [Na·2.5H_2_O][Cl_6_-FESANE]

IR (KBr): *ν* [cm^–1^] = 3576 (vs, *ν*_s_(H_2_O)), 3039 (vs, *ν*_s_(C_c_–H)), 2567 (vs, *ν*_s_(B–H)), 1612 (vs, *ν*(H_2_O)). ^1^H NMR: *δ* [ppm] = 39.20 (br s, 4H, C_c_–*H*), –6.59, –11.8, –15.54, –24.10 (br s, 12H, B–*H*_*t*_). ^11^B NMR: *δ* [ppm] = 126.7, 104.4, 45.8, 38.4, 29.4, 7.6, 3.3, –11.6, –28.8, –70.3, –334.0, –351.6, –412.3, –423.4, –498.1, –510.5, –542.8. MALDI-TOF MS: *m*/*z* (%) = 493.16 [M – Cl, 20.8%]^–^, 527.13 [M, 100%]^–^, 562.10 [M + Cl, 14.0%]^–^. UV-vis in H_2_O at 10^–4^ M: 247 (0.212), 295 (0.404), 335 (0.384), 607 (0.003) nm.

### Interaction with DNA

#### Melting experiments

Melting curves were performed by monitoring the DNA absorbance at 260 nm with heating in a UV-visible spectrophotometer in conjunction with a thermostated bath. The measurements were carried out in 0.1 M PB pH 7.0 solution. The temperature inside the cuvette was determined with a platinum thermocouple and was increased over the range 30–90 °C at a heating rate of 1 °C min^–1^. The melting temperature, *T*_m_, was obtained from the mid-point of the hyperchromic transition.

#### Circular dichroism measurements

CD spectra of CT-dsDNA in the presence and absence of Na[FESANE] and Na[Cl_6_-FESANE] were obtained using a J715 spectropolarimeter at 25 °C along with a 0.1 cm pathlength cuvette. The spectra were recorded in the 220–320 nm region for 80 μM CT-dsDNA in the presence of 8 μM of Na[FESANE] and Na[Cl_6_-FESANE] solutions, respectively.

#### Electrochemical studies

Cyclic voltammetry and differential pulse voltammetry measurements were carried out in 0.1 M PB pH 7.0 solution. The parameters used in the DPV measurements were: scan rate 10 mV s^–1^; pulse amplitude 50 mV; pulse width 0.2 s. All the differential pulse voltammograms presented were baseline-corrected using the application included in GPES version 4.9 software. No changes in the current or potential values were observed relative to the data obtained without such application.

The ratio of the equilibrium surface-binding constants (*K*_ox_/*K*_red_) was estimated from the *E*^0^′ shifts (Δ*E*^0^′) of the DNA/Au electrodes with respect to *E*^0^′ of the unmodified Au electrodes and using eqn (1) according to the model of Bard and Carter:^40^1
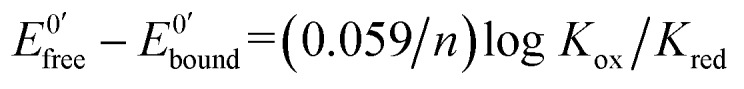
where 
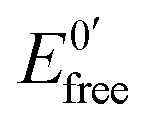
 and 
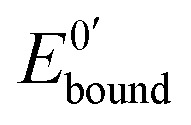
 represent the formal potentials of the metallacarborane in solution and bound to DNA and *K*_ox_ and *K*_red_ are the respective equilibrium binding constants to DNA for the oxidized and reduced forms of the metallacarborane.

Electrochemical experiments in the presence of DNA were performed at a fixed concentration of metallacarboranes (1.0 mM), varying the concentration of CT-dsDNA (from 0 to 500 μM).

### Biosensor development

#### Pretreatment of the AuSPE

The AuSPEs were electrochemically activated by placing a 50 μl drop of 0.1 M H_2_SO_4_ solution on their surface. Then the potential was cycled (10 times) from –0.200 V to 1.20 V at 100 mV s^–1^ and the electrodes were washed with deionized water.

#### Immobilization of DNA on AuSPE

For CT-dsDNA or CT-ssDNA immobilization, the conditioned AuSPE was immediately modified by transferring 10 μl of 2.0 mM CT-dsDNA or CT-ssDNA solution onto its surface followed by air-drying. Afterwards, the electrode was soaked in sterilized water for 30 min and rinsed with water to remove any un-adsorbed DNA. These modified electrodes are denoted CT-dsDNA/AuSPE and CT-ssDNA/AuSPE in the text, respectively.

In the case of DNA oligonucleotides, 10 μl of a 40 μM thiolated synthetic sequence (SH-HP1, SH-WT_11_ or SH-WT_12_) were transferred onto a clean AuSPE. Afterwards, the electrode was kept for 24 hours at room temperature. Then it was soaked in sterile water for at least 30 min.

#### Denaturation of PCR DNA samples

PCR samples were denatured immediately before use by heating up to 100 °C within 20 minutes followed by rapid cooling in an ice bath.^48^

#### Hybridization and detection

The AuSPEs modified with the capture probe were subsequently hybridized (1 h, 40 °C) with 10 μl of the analyte: denatured PCR samples, 5.0 ng μl^–1^ wild type (WT_11_, WT_12_) or mutated (SNPG542X or MUTF508del) forms, or synthetic oligonucleotides (20 μM of complementary HP2C or HP2SNP sequences). After the hybridization, the modified electrodes were immersed in 0.1 M PB pH 7.0 solution containing 1.0 mM Na[FESANE] or Na[Cl_6_-FESANE] and the potential was cycled (100 times) at 100 mV s^–1^ between –0.6 V and 0.0 V and –0.0 V to 0.7 V, respectively. Then the electrodes were rinsed with sterile water, placed in 0.1 M PB pH 7.0 solution, and differential pulse voltammograms were immediately recorded.

## Conclusions

The synthesis and characterization of two different metallacarboranes, [Na·2.5H_2_O] [3,3-Fe(1,2-*closo*-C_2_B_9_H_11_)_2_] and [Na·2.5H_2_O][3,3-Fe-(8,9,12-Cl_3_-1,2-*closo*-C_2_B_9_H_8_)_2_], and their interactions with CT-dsDNA and CT-ssDNA were thoroughly investigated. From these studies, it is clear that there is a strong interaction between these anionic metallacarboranes and DNA, and that they can be accumulated at a surface-immobilized DNA layer of screen-printed electrodes. Using these findings and the fact that both compounds are electrochemical active as a starting point, they have been employed as redox indicators of DNA hybridization in the development of electrochemical DNA biosensors as screening methods for the detection of gene mutations. A wide range of applications of this type of biosensor is foreseen because their fabrication and measuring protocols are simple, economical, and non-time consuming. In addition, these biosensors can be easily adapted for the detection of mutations, as a method having immense potential for greatly simplifying the development of point-of-care molecular diagnostic analysis systems.

## Supplementary Material

Supplementary information
