# Supplementary material for: Metallacarboranes as tunable redox potential electrochemical indicators for screening of gene mutation
Source: Chem Sci. 2016 Jun 8;7(9):5786–97. doi: 10.1039/c6sc01567k (PMC6022041; doi:10.1039/c6sc01567k)
Supplement: Supplementary file 1 [file SC-007-C6SC01567K-s001.pdf]

Electronic Supplementary Material (ESI) for Chemical Science.
This journal is © The Royal Society of Chemistry 2016

Supporting Information

Metallacarboranes as tunable redox potential electrochemical indicators for screening of gene mutation.

Tania García-Mendiola [a,b], Victoria Bayon-Pizarro [a], Adnana Zaulet[c], Isabel Fuentes [c], Félix Pariente [a,b] , Francesc Teixidor [c] , Clara Viñas [*c] and Encarnación Lorenzo [*a,b]

[a] Departamento Química Analítica y Análisis Instrumental, Universidad Autónoma de Madrid, Spain.

[b] Instituto Madrileño de Estudios Avanzados (IMDEA) Nanociencia

[c] Institut de Ciència de Materials de Barcelona (ICMAB-CSIC).Campus UAB, 08193 Bellaterra, Barcelona, Spain

[*]e mail: encarnacion.lorenzo@uam.es, clara@icmab.es

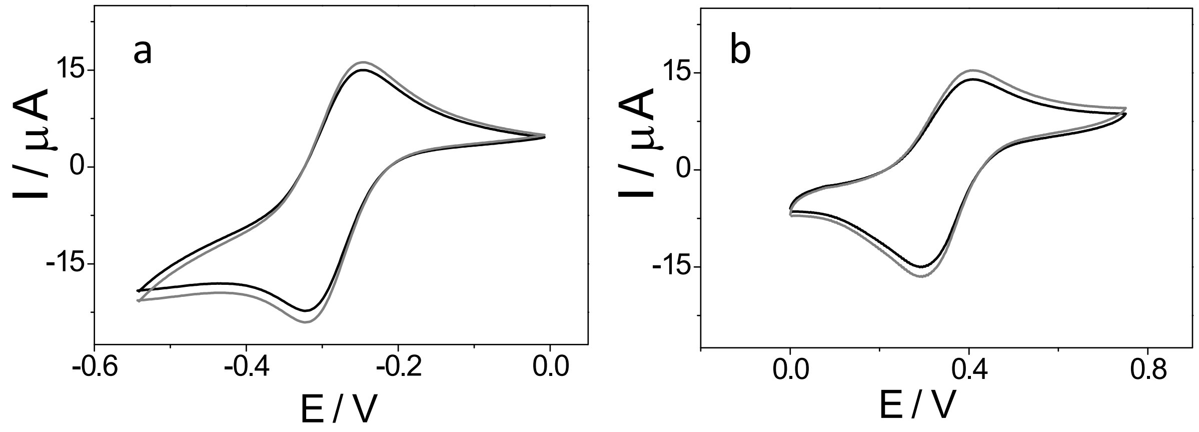

Figure 1 SI. Cyclic voltammograms of 1.0 mM of Na[FESANE] (a) and Na[Cl_6-FESANE] (b) in 0.1 M PB pH 7.0 solution (black curve) and 0.1 M PB pH 7.0 solution + 0.4 M NaCl (grey curve) at a AuSPE. Scan rate: 100 mV s^{-1}.

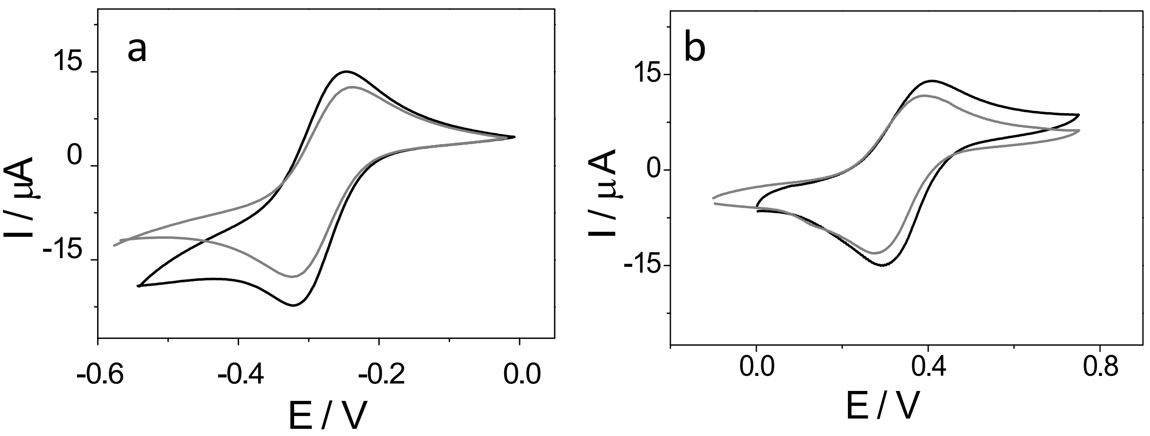

Figure 2 SI. Cyclic voltammograms of 1.0 mM of Na[FESANE] (a) and Na[Cl_6-FESANE] (b) in absence (black curve) and in presence of CT-dsDNA 500 μM (grey curve) in 0.1 M PB pH 7.0 solution at a AuSPE. Scan rate: 100 mV s^{-1}.

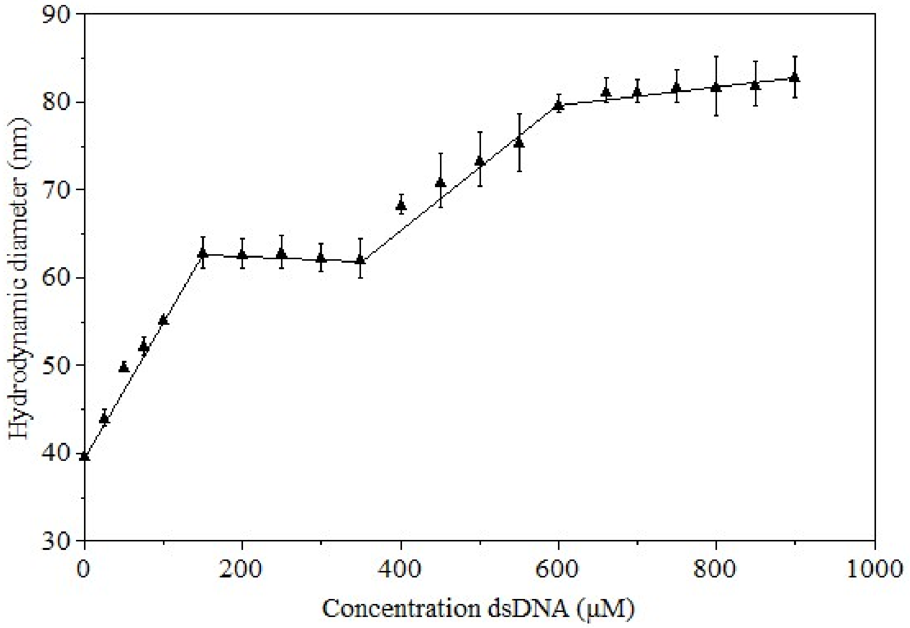

3 SI. Graphical representation of the hydrodynamic diameter (nm) of Na [FESANE] aggregates in aqueous 1.0 mM solution measured by DLS versus increasing concentrations of CT-dsDNA in water in the concentration range from 0 to 900 µM.

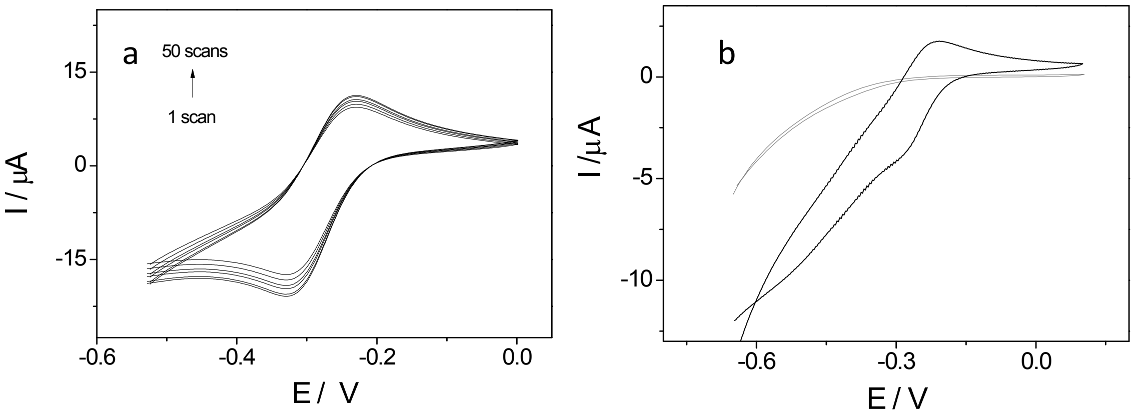

Figure 4 SI. a) Consecutive cyclic voltammograms of 1.0 mM Na[FESANE] in 0.1 M PB pH 7.0 solution at a CT-dsDNA/AuSPE. b) Cyclic voltammogram in 0.1 M phosphate buffer pH 7.0 solution at a CT-dsDNA/AuSPE before (grey curve) and after the accumulation of Na[FESANE] by consecutive cyclic scans (black curve). Scan rate: 100 mV s^{-1}.

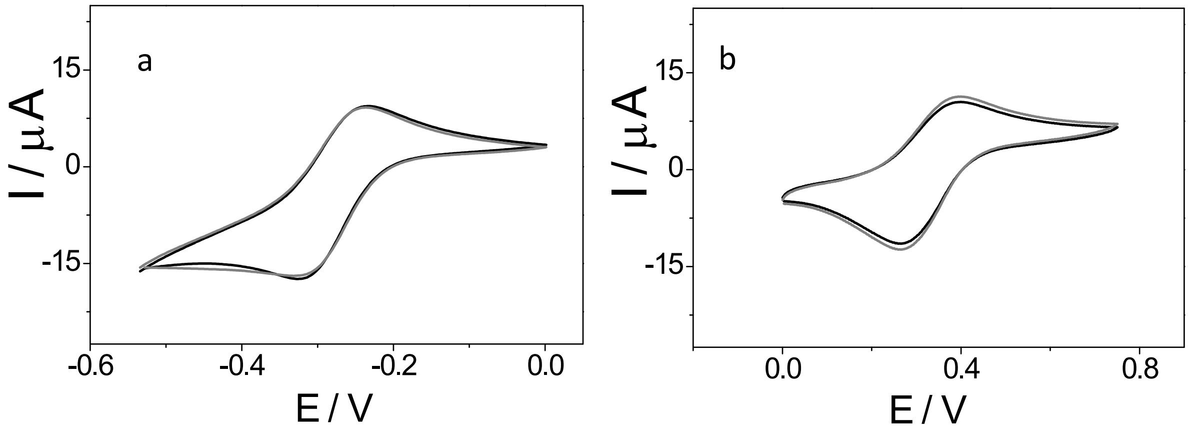

Figure 5 SI. Cyclic voltammograms of 1.0 mM Na[FESANE] (a) and Na[Cl_6-FESANE] (b) in 0.1 M PB pH 7.0 solution (black curve) and 0.1 M PB pH 7.0 solution + 0.4 M NaCl (grey curve) at a CT-dsDNA/AuSPE. Scan rate: 100 mV s^{-1}.
